# Trajectories of olfactory identification preceding incident mild cognitive impairment and dementia: a longitudinal study

**DOI:** 10.1016/j.ebiom.2023.104862

**Published:** 2023-10-28

**Authors:** Jie Guo, Abigail Dove, Jiao Wang, Erika J. Laukka, Ingrid Ekström, Michelle M. Dunk, David A. Bennett, Weili Xu

**Affiliations:** a Department of Nutrition and Health, China Agricultural University, Beijing, China; b Aging Research Center, Department of Neurobiology, Care Sciences and Society, Karolinska Institutet, Stockholm, Sweden; c Department of Epidemiology and Biostatistics, School of Public Health, Tianjin Medical University, Tianjin, China; d Tianjin Key Laboratory of Environment, Nutrition and Public Health, Tianjin, China; e Center for International Collaborative Research on Environment, Nutrition, and Public Health, Tianjin, China; f Department of Epidemiology, College of Preventive Medicine, The Army Medical University (Third Military Medical University), Chongqing, China; g Stockholm Gerontology Research Center, Stockholm, Sweden; h Rush Alzheimer’s Disease Center, Rush University Medical Center, Chicago, IL, USA

**Keywords:** Olfactory identification, Trajectory, Mild cognitive impairment, Dementia, Brain pathology

## Abstract

**Background:**

The pattern of olfactory identification change in the early phases of dementing disorders is unclear. We aimed to assess olfactory identification trajectories preceding incident mild cognitive impairment (MCI) and dementia and explore the role of brain pathologies in these trajectories.

**Methods:**

Within the Rush Memory and Aging Project, 1318 dementia-free older adults were followed annually for up to 11 years. Olfactory identification was assessed using the Brief Smell Identification Test annually. Of 900 cognitively intact participants, incident MCI and dementia were diagnosed following standard criteria. Over follow-up, 518 participants died and underwent brain autopsies for neuropathological assessment. Data were analyzed using mixed-effect models with backward timescales.

**Findings:**

Compared to participants who remained cognitively intact, olfactory identification declined faster among those who developed MCI (β −0.09 [95% CI −0.13, −0.05]), leading to a significantly lower olfactory identification starting from five years preceding MCI diagnosis (mean difference at year −5: −0.39 [−0.71, −0.07]). Among participants with incident MCI, olfactory identification declined faster in those who developed dementia compared to those who did not (β −0.19 [−0.36, −0.01]), leading to a significantly lower olfactory identification starting from three years preceding dementia diagnosis (mean difference at year −3: −0.95 [−1.67, −0.23]). A faster decline in olfactory identification was associated with higher burdens of global Alzheimer’s disease pathology, neurofibrillary tangles, and amyloid beta load.

**Interpretation:**

Olfactory identification declined faster preceding dementia disorders and Alzheimer’s pathology may underlie these faster declines.

**Funding:**

This study was funded by the 10.13039/100000002National Institutes of Health (R01AG17917) and 10.13039/501100004359Swedish Research Council (2021-01647).

**Research in context**

**Evidence before this study**

Olfactory dysfunction has been linked to an increased risk of mild cognitive impairment (MCI) or dementia and has been associated with an accelerated progression from MCI to dementia. In this context, questions remain about how olfactory identification differs between normal aging individuals and those with preclinical dementia. Previous studies on the relationship between olfactory identification and brain pathologies have shown inconsistent results. Some studies have linked amyloid burden with poorer olfactory identification among older adults, although others have not. Conversely, neurofibrillary tangles have been consistently associated with olfactory dysfunction. Thus, the extent to which Alzheimer’s and non-Alzheimer’s disease pathologies might underlie long-term olfactory identification change is unclear.

**Added value of this study**

This study shows that a faster decline in olfactory identification may occur in the early stage of dementing disorders, though the estimated single time-point olfactory identification still falls within the normal range. A high burden of global Alzheimer’s disease pathology, especially neurofibrillary tangles, may underlie the accelerated decline in olfactory identification during the early stage of dementia.

**Implications of all the available evidence**

Monitoring changes in olfactory identification may be a strategy for early detection of individuals at a high risk of developing MCI or dementia, even among individuals whose olfactory identification falls within the normal range. Future studies are warranted to explore whether interventions or treatments can prevent or delay the progression of dementing disorders when administered early enough, such as among individuals with a rapid olfactory decline but no apparent cognitive impairment.

## Introduction

Globally, over 50 million people had dementia in 2019, and this number is projected to almost triple by 2050.^1^ Effective prevention for dementia remains elusive, albeit a widespread interest.^2^ Pathophysiological processes underlying dementia may begin a decade or more before the clinical diagnosis of dementia or mild cognitive impairment (MCI, a reversible intermediate state between normal cognitive function and dementia).^3^ Hence, the development of reliable and practical screening tools to identify individuals in the preclinical stage of dementia is crucial. Such tools will enable the implementation of interventions and treatments that may inhibit pathological processes before symptoms manifest and cognitive decline becomes inevitable.

Olfactory dysfunction has been identified as a predictor of MCI^4^, ^5^, ^6^, ^7^ and dementia,^8^, ^9^, ^10^ and has been associated with accelerated progression from MCI to dementia.^6^^,^^11^ Brain areas involved in olfaction, such as the entorhinal cortex and hippocampus,^12^ appear to be the first impacted by Alzheimer’s disease (AD) pathologies.^13^^,^^14^ However, the relationship between olfactory identification and brain pathologies is not fully understood. Amyloid burden has been reported to be associated with poorer olfactory identification among older adults,^15^ although this has not been replicated in other studies.^5^^,^^16^, ^17^, ^18^, ^19^ Previous findings have consistently shown a positive association between neurofibrillary tangles and olfactory dysfunction.^5^^,^^17^^,^^20^

In our previous studies based on the Rush Memory and Aging Project (MAP), impaired olfactory identification was associated with cognitive impairment,^21^, ^22^, ^23^ and rapid decline of olfactory identification was associated with an increased risk of MCI or dementia.^24^ However, our understanding of the natural history of olfactory identification in the early phases of dementing disorders is limited, and questions remain regarding how olfactory identification differs between normal aging and preclinical dementia. Moreover, we previously found that higher levels of amyloid deposits and neurofibrillary tangles and the presence of Lewy bodies were associated with olfactory identification.^25^^,^^26^ However, evidence about other non-AD pathologies, such as cerebral vascular pathologies, is limited. Our previous studies mainly considered single time-point olfactory identification.^25^^,^^26^ Here, we therefore extended our prior work by investigating long-term changes in olfactory identification in relation to cognitive outcomes and AD and non-AD brain pathologies.

In this study, using a community-based cohort study with repeatedly measured olfactory identification and autopsy pathological data, we aimed to 1) investigate how olfactory identification evolves before the onset of MCI among cognitively intact individuals, 2) assess olfactory identification trajectories before dementia diagnosis among those with incident MCI, and 3) explore the associations between olfactory identification trajectories and brain pathologies.

## Methods

### Study design and participants

The Rush MAP is an ongoing longitudinal cohort study recruiting older adults from senior and subsidized housing, continuous care retirement communities, social service agencies, church groups, and individual homes in northeastern Illinois and the Chicago area.^27^ At enrolment and thereafter, all participants underwent a comprehensive clinical assessment, including neurological examination, cognitive tests, and medical history.^27^ Information on socio-demographic factors (age, sex, race/ethnicity, and education), physical activity, medical conditions (diabetes, hypertension, and vascular diseases), and apolipoprotein E (*APOE*) alleles was collected at study entry (Detailed information shown in Supplementary Text S1).

**Fig. 1:**
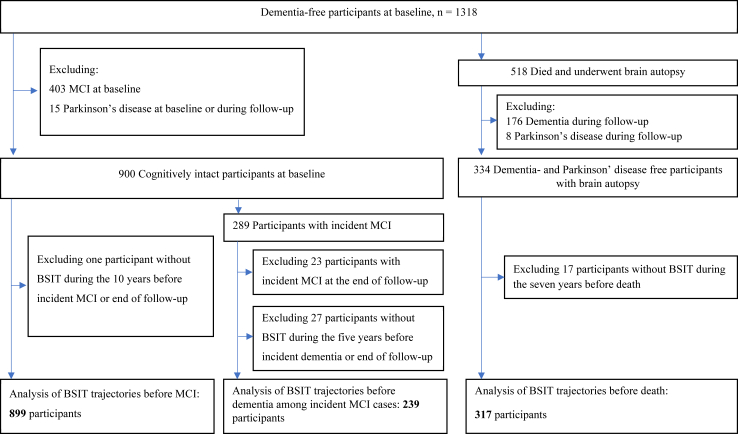
Flowchart of the study population. Abbreviations: MCI, mild cognitive impairment; BSIT, the brief smell identification test.

Enrolment for Rush MAP has been ongoing since 1997. Annual olfaction testing began in 2011, so this study uses 2011 as the analytical baseline. A total of 1318 dementia-free participants were enrolled at baseline and followed-up annually through 2022 (maximum follow-up of 11 years). We excluded 403 individuals with prevalent MCI at baseline and 15 with Parkinson’s disease at baseline or during follow-up, leaving 900 cognitively intact participants for the analysis of olfactory identification trajectories before MCI. 289 of these 900 participants developed incident MCI and were included for the analysis of trajectories before dementia. Over the follow-up, 518 participants died and underwent brain autopsy. We further excluded 176 participants with dementia and eight with Parkinson’s disease before death, leaving 334 for the analyses of trajectories in relation to brain pathologies (Fig. 1).

### Assessment of olfactory identification

The 12-item Brief Smell Identification Test (BSIT) was used to assess the ability to identify familiar odors.^28^ For each item, a microcapsule containing a familiar odor was scratched with a pencil and placed under the nose of the participant, who was asked to identify the smell based on 4 possible options. A maximum of two missing responses was allowed, with a score of 0.25 assigned to each missing item.^19^ If responses to 3 or more items were missing, data on the test were considered missing. The total score reflected the number of odors that were correctly recognized (range from 0 to 12). Normal olfactory identification is defined as a BSIT score > 8 and impaired olfactory identification as BSIT ≤ 8.^28^

### Assessment of MCI and dementia

To evaluate clinical diagnoses of dementia and MCI, a standardized and structured process was utilized that incorporated computerized cognitive test scoring, clinical evaluation by a neuropsychologist, and diagnostic classification by a clinician.^29^ The diagnostic criteria used for dementia was based on the joint working group of the National Institute of Neurological and Communicative Disorders and Stroke and the Alzheimer's Disease and Related Disorders Association.^29^ A diagnosis of MCI was rendered for participants who did not meet the criteria for dementia but were judged by a neuropsychologist to have objective impairment in cognitive function based on data from cognitive tests and considering age and education level.^29^

### Assessment of brain pathologies

Data on post-mortem AD and non-AD pathology were collected in MAP according to a standard protocol and detailed information about autopsy procedures has been described previously.^25^ Global AD pathology (including neuritic plaques, diffuse plaques, and neurofibrillary tangles), amyloid beta load, and cerebral vascular disease pathology (including atherosclerosis, arteriolosclerosis, and cerebral amyloid angiopathy) were tertiled. Lewy body pathology and chronic gross- and micro-infarcts were dichotomized as present vs. absent.

### Statistical analyses

Baseline characteristics of the study population with or without brain autopsy were compared using chi-square tests for categorical variables and the *t-test* or Wilcoxon rank-sum tests for continuous variables.

To examine olfactory identification trajectories before MCI, we used linear mixed-effect models with backward timescales. The assumptions of linear-mixed effect model, including linearity, homoscedasticity for the residuals, and normality of the residuals, were tested and no violations were observed. In the backward timescale, year 0 was the year of MCI diagnosis or the year corresponding to the end of follow-up (for participants who remained cognitively intact), year −1 was one year preceding year 0, and so on. MCI status, time (i.e., the year corresponding olfactory data was collected), and their interaction were included in the model to test for differences in olfactory identification by MCI status (coded as 0 for cognitively intact and 1 for incident MCI). The random effects included random intercept and slope to reflect individual differences in olfactory identification at year 0 and its change over time. The difference in olfactory identification between cognitively intact participants and those who developed MCI was estimated for each year preceding year 0, with a negative value indicating lower olfactory identification among the MCI group. All analyses were adjusted for age at time 0, sex, and education, and the interaction terms between these variables and time when *P*-value for interactions < 0.05. Similarly, among participants with incident MCI, we analyzed BSIT trajectories before dementia diagnosis, using the year of incident dementia or the last follow-up as year 0. The reference group was participants with incident MCI who did not develop dementia during the follow-up. The length of the backward timescales was decided based on the sample size for each time point.

To explore the potential role of brain pathologies in olfactory identification trajectories preceding dementia, we examined olfactory identification trajectories by brain pathologies among dementia-free participants, using the year of death/brain autopsy as year 0. To assess the independent role of brain pathologies, we included brain pathologies with significant results in the same model.

In the sensitivity analyses, we included participants with ≥3 repeated BSIT measurements. Moreover, stratified analyses by sex and *APOE* genotype were performed. We further conducted analyses excluding participants diagnosed with other types of dementia to explore the trajectories of olfactory identification preceding Alzheimer’s disease dementia diagnosis. Additionally, missing values for olfactory identification were imputed using the fully conditional specification method for participants without olfactory identification after MCI diagnosis and before dementia diagnosis or end of follow-up (N = 27). Estimates from 10 iterations were pooled according to Rubin’s rules.^30^ Statistical analyses were performed using SAS 9.4 (SAS Institute, Cary, NC). All *P*-values were two-sided, and we defined statistical significance as *P* < 0.05. To account for the multiple testing and comparisons of differences in estimated BSIT, a simulation-based approach combined with a step-down fashion was used to calculate adjusted *P*-values and confidence intervals (CIs).^31^ Data were analyzed from October 2022 to March 2023.

### Ethics

This study received approval from the Institutional Review Board at Rush University Medical Center (L99032481-CR10 and L86121802-CR12) and adhered to the ethical standards in the 1964 Declaration of Helsinki and its subsequent amendments. Before enrolment, all individuals provided informed consent and completed an Anatomical Gift Act for organ donation. Moreover, participants signed a repository consent form, granting permission for their data to be shared. This study followed the Strengthening the Reporting of Observational Studies in Epidemiology (STROBE) reporting guidelines.

### Role of the funding source

The funding source had no role in the design and conduct of the study; the collection, management, analysis, or interpretation of the data; the preparation, review, or approval of the manuscript; or the decision to submit the manuscript for publication.

## Results

### Baseline characteristics of study population

Of the 1318 baseline dementia-free participants, the mean age (standard deviation [SD]) was 81.0 (7.8) years, 995 (75.5%) were females, and 1088 (82.5%) were White. Of the 518 participants who died and underwent brain autopsies during follow-up, the mean age (SD) was 85.3 (6.0) years, 384 (74.1%) were females, and 491 (94.8%) were White. Compared to participants who were alive at the end of follow-up, those who underwent brain autopsies were more likely to be older, White, less educated, less physically active, have hypertension, be diagnosed with vascular diseases, and have a lower level of olfactory identification (*P* < 0.05 for all) (Table 1).

**Table 1:** Characteristics of the dementia-free population at baseline.

Characteristic	Total (n = 1318)	Without brain autopsy (n = 800)	With brain autopsy (n = 518)	*P* value
Age at baseline (y), mean (SD)	81.0 (7.8)	78.2 (7.5)	85.3 (6.0)	<0.001
Sex, n (%)				0.355
Female	995 (75.5)	611 (76.4)	384 (74.1)	
Male	323 (24.5)	189 (23.6)	134 (25.9)	
Race/ethnicity, n (%)				<0.001
White	1088 (82.5)	597 (74.6)	491 (94.8)	
Black or African American	58 (4.4)	49 (6.1)	9 (1.7)	
Other	10 (0.8)	10 (1.2)	NA	
Education (y), mean (SD)	15.4 (3.3)	15.5 (3.5)	15.1 (2.9)	0.029
Physical activity (h/week), median (IQR)	2.5 (0.9, 4.8)	2.9 (1.0, 5.7)	2.3 (0.7, 4.1)	<0.001
BSIT, mean (SD)	9.2 (2.3)	9.6 (2.1)	8.5 (2.5)	<0.001
Diabetes, n (%)	170 (12.9)	105 (13.1)	65 (12.5)	0.780
Hypertension, n (%)	905 (68.7)	530 (66.3)	375 (72.4)	0.014
Any vascular diseases^a^, n (%)	334 (25.3)	149 (18.6)	185 (35.7)	<0.001
Apolipoprotein E ε4 carriers, n (%)	225 (17.1)	130 (16.3)	95 (18.3)	0.193

Missing data: 280 for Apolipoprotein E ε4 status; 162 for race/ethnicity, 2 for hypertension.

Abbreviations: SD, standard deviation; IQR, interquartile range; BSIT, the Brief Smell Identification Test.

a Vascular diseases included claudication, stroke, heart conditions (i.e., heart attack or coronary, coronary thrombosis, coronary occlusion, or myocardial infarction), and congestive heart failure.

### Olfactory identification trajectory before incident MCI

**Fig. 2:**
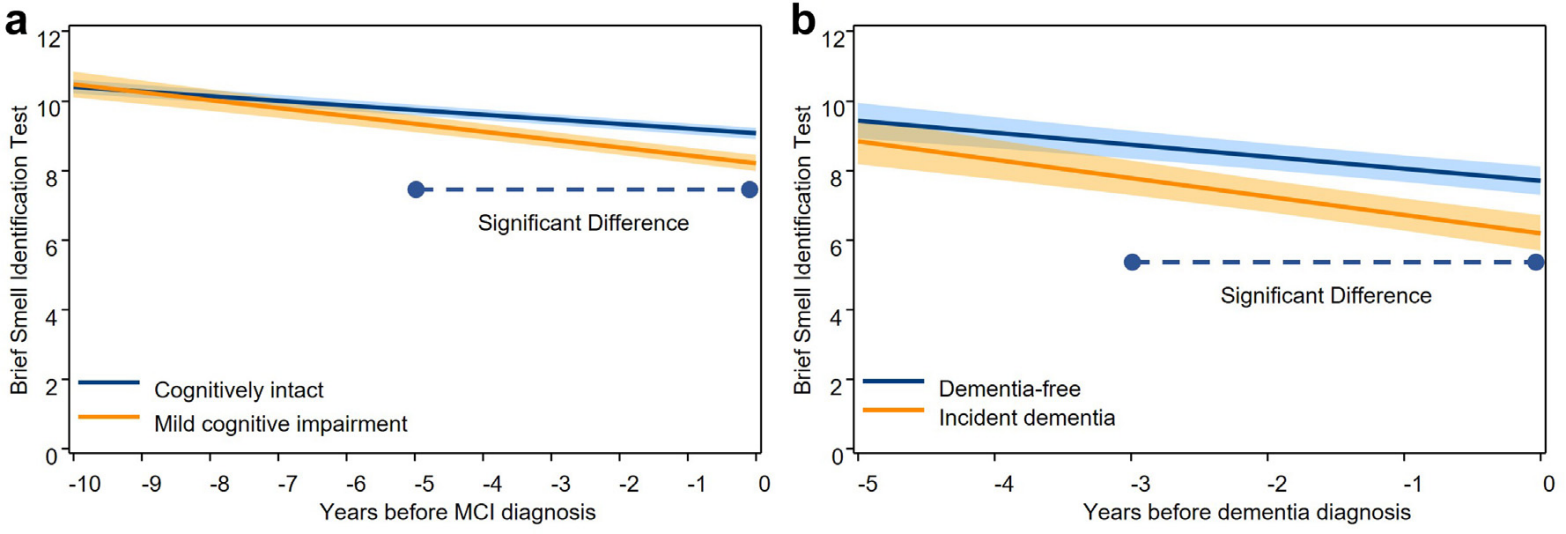
Trajectories of Brief Smell Identification Test (BSIT) in the 10 years before mild cognitive impairment (MCI) diagnosis (a) and in the 5 years before dementia diagnosis (b). The figure represents marginal effects of MCI or dementia on trajectories of BSIT, adjusted for age at time 0, sex, and education. The band represents the 95% confidence interval of estimated mean BSIT. For the multiple testing and comparisons, the significant differences were defined using adjusted *P* value < 0.05 calculated from a simulation-based approach combined with a step-down fashion. For BSIT trajectories before MCI diagnosis (a), β coefficient for MCI × time is −0.09 (95% CI −0.13, −0.05; *P* < 0.001). For BSIT trajectories before dementia diagnosis (b), β coefficient for dementia × time is −0.19 (95% CI −0.36, −0.01; *P* = 0.040).

The trajectory of olfactory identification differed between participants who developed MCI (n = 289) and those who remained cognitively intact (n = 610), such that those with incident MCI had an earlier and more pronounced decline (β [95% CI] for MCI × time: −0.09 [−0.13, −0.05], *P* < 0.001) (Fig. 2a). Participants with incident MCI had a lower olfactory identification about five years preceding diagnosis compared to cognitively intact participants (mean difference at year −5 [95% CI]: −0.39 [−0.71, −0.07], *P* = 0.007) (Table 2). The difference in olfactory identification became larger thereafter (mean difference at year 0 [95% CI]: −0.85 [−1.17, −0.53], *P* < 0.001). If using a BSIT score ≤ 8 to define impaired olfactory identification,^28^ the estimated mean of BSIT among participants who developed MCI was still within the normal range even at the MCI diagnosis. Similar results were observed when including participants with at least three repeated BSIT (Supplementary Fig. S1 and Table S1).

**Table 2:** Differences in Brief Smell Identification Test (BSIT) between mild cognitive impairment (MCI) cases and cognitively intact participants in the 10 years before MCI diagnosis in cognitively intact sample.

Year	No. of cognitively intact participants	No. of MCI participants	Incident MCI vs. cognitively intact	
			Difference in mean (95% CI)	*P*-value
−10	118	11	0.06 (−0.42, 0.54)	0.790
−9	178	23	−0.03 (−0.47, 0.41)	0.875
−8	213	36	−0.12 (−0.52, 0.28)	0.527
−7	244	56	−0.21 (−0.58, 0.16)	0.222
−6	278	72	−0.30 (−0.64, 0.04)	0.056
−5	318	97	−0.39 (−0.71, −0.07)	0.007
−4	393	117	−0.48 (−0.79, −0.18)	0.001
−3	454	169	−0.58 (−0.87, −0.28)	<0.001
−2	499	202	−0.67 (−0.96, −0.37)	<0.001
−1	529	267	−0.76 (−1.06, −0.46)	<0.001
0	610	289	−0.85 (−1.17, −0.53)	<0.001

Difference in mean was calculated as the mean of BSIT in participants with MCI minus that in those cognitively intact. Negative value means that olfactory identification was lower in participants with MCI. Model was adjusted for age at time 0, sex, and education.

### Olfactory identification trajectory before incident dementia

Olfactory identification declined faster among participants who went on to develop dementia (n = 81) compared to those who did not (n = 158) (β [95% CI] for dementia × time: −0.19 [−0.36, −0.01], *P* = 0.040) (Fig. 2b). Compared with dementia-free participants, a lower olfactory identification was observed in those with incident dementia starting from three years before diagnosis (mean difference at year −3 [95% CI]: −0.95 [−1.67, −0.23], *P* = 0.003) (Table 3). This difference became larger thereafter (mean difference at year 0 [95% CI]: −1.51 [−2.25, −0.77], *P* < 0.001). Similar results were observed when including participants with at least three repeated BSIT (Supplementary Fig. S1 and Table S2). Similar results were also observed when excluding participants diagnosed with other types of dementia but not Alzheimer’s disease dementia. Olfactory identification declined faster among participants who went on to develop Alzheimer’s disease dementia (n = 75) compared to those who did not (n = 158) (β [95% CI] for dementia × time: −0.18 [−0.36, −0.003], *P* = 0.040), leading to a lower olfactory identification starting from three years before diagnosis (mean difference at year −3 [95% CI]: −0.89 [−1.62, −0.17], *P* = 0.007). Moreover, we obtained a similar result after including 27 participants with imputed olfactory identification (β [95% CI] for dementia × time: −0.16 [−0.34, 0.01], *P* = 0.071).

**Table 3:** Differences in Brief Smell Identification Test (BSIT) between incident dementia cases and participants who remained mild cognitive impairment (MCI) non-dementia ones in the 5 years before dementia diagnosis in the incident MCI sample.

Year	No. of non-dementia participants	No. of dementia participants	Incident dementia vs. remain MCI	
			Difference in mean (95% CI)	*P*-value
−5	35	17	−0.58 (−1.55, 0.38)	0.164
−4	52	28	−0.77 (−1.59, 0.06)	0.038
−3	78	38	−0.95 (−1.67, −0.23)	0.003
−2	88	57	−1.14 (−1.80, −0.48)	<0.001
−1	106	71	−1.33 (−2.00, −0.65)	<0.001
0	158	81	−1.51 (−2.25, −0.77)	<0.001

Difference in mean was calculated as the mean of BSIT in participants with dementia minus that in those remain MCI. Negative value means that olfactory identification was lower in participants with dementia. Model was adjusted for age at time 0, sex, and education.

### Olfactory identification trajectories in relation to brain pathologies

**Fig. 3:**
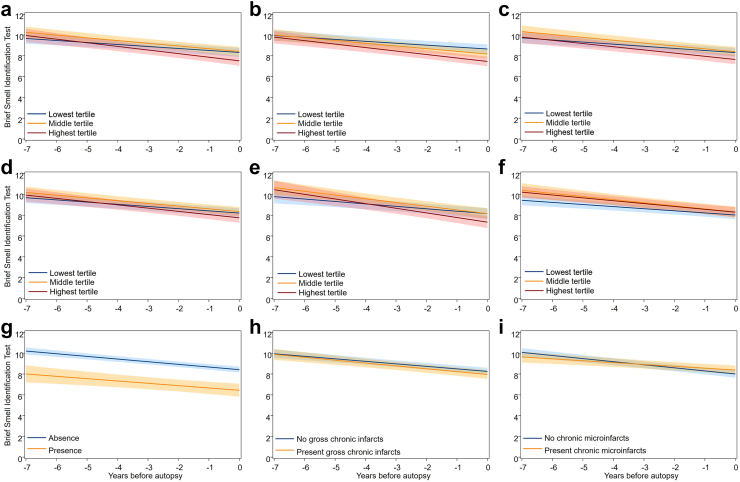
Trajectories of Brief Smell Identification Test (BSIT) among dementia-free participants in the 7 years before brain autopsy. The figure represents marginal effects of brain pathologies, including the burden of global Alzheimer’s disease pathology (a), neurofibrillary tangles (b), neuritic plaque (c), diffuse plaque (d), amyloid beta load (e), vascular disease pathology (f), Lewy body pathology (g), gross chronic infarcts (h), and chronic microinfarcts (i), on trajectories of BSIT, adjusted for age at time 0, sex, and education. The band represents the 95% confidence interval of estimated mean BSIT. Higher burdens of global AD pathology (a; β [95% CI] for pathology × time, highest vs. lowest: −0.16 [−0.28, −0.04], *P* = 0.011), neurofibrillary tangles (b; highest vs. lowest: −0.15 [−0.26, −0.04], *P* = 0.009), and amyloid beta load (e; highest vs. lowest: −0.21 [−0.38, −0.04], *P* = 0.014) were associated with faster declines in olfactory identification.

Fig. 3 shows the trajectories of olfactory identification in the seven years before death across different levels of brain pathologies. Higher burdens of global AD pathology (β [95% CI] for pathology × time, highest vs. lowest: −0.16 [−0.28, −0.04], *P* = 0.011), neurofibrillary tangles (highest vs. lowest: −0.15 [−0.26, −0.04], *P* = 0.009), and amyloid beta load (highest vs. lowest: −0.21 [−0.38, −0.04], *P* = 0.014) were associated with faster declines in olfactory identification (Fig. 3a, b, e, and Supplementary Table S3). After including neurofibrillary tangles and amyloid beta load in the model simultaneously, the association was slightly changed for neurofibrillary tangles (highest vs. lowest: −0.17 [−0.34, 0.005], *P* = 0.057) and largely altered to non-significant for amyloid beta (highest vs. lowest: −0.14 [−0.33, 0.05], *P* = 0.160). Moreover, the presence of Lewy body pathology was associated with lower olfactory identification starting from at least seven years before death (mean difference at year −7 [95% CI]: −2.20 [−3.21, −1.19], *P* < 0.001) (Fig. 3g and Supplementary Table S4), though olfactory identification change was not significantly associated with Lewy body pathology (0.03 [−0.10, 0.16], *P* = 0.637).

We did not find statistically significant associations of olfactory identification change with neuritic plaques, diffuse plaques, vascular disease pathology, or gross infarcts (Fig. 3c, d, f and h). However, chronic microinfarcts were associated with a slower decline in olfactory identification (0.12 [0.02, 0.21], *P* = 0.014) (Fig. 3i and Supplementary Table S3).

### Supplementary results

Among both females and males, those with incident MCI had a more pronounced decline (β [95% CI] for MCI × time: in females −0.06 [−0.11, −0.01], *P* = 0.020; in males −0.19 [−0.28, −0.11], *P* < 0.001) (Supplementary Fig. S2a and b). Compared to cognitively intact participants, those with incident MCI had a lower olfactory identification about five years prior to diagnosis in females (mean difference at year −5 [95% CI]: −0.42 [−0.79, −0.05], *P* = 0.014) and about three years prior to diagnosis in males (mean difference at year −3 [95% CI]: −0.69 [−1.29, −0.10], *P* = 0.014) (Supplementary Table S5). Among *APOE* ε4 carriers, those with incident MCI had a more pronounced decline (β [95% CI] for MCI × time: −0.10 [−0.19, 0.00], *P* = 0.051) (Supplementary Fig. S3a), leading to a significantly lower olfactory identification beginning three years before diagnosis (Supplementary Table S6). A similar result was observed among *APOE* ε4 non-carriers (β [95% CI] for MCI × time: −0.08 [−0.13, −0.03], *P* < 0.001) (Supplementary Fig. S3b), with a significantly lower olfactory identification beginning three years before diagnosis (Supplementary Table S6).

## Discussion

In this prospective cohort study of older adults with annually assessed olfactory identification, we found that 1) those who developed MCI had a faster decline in olfactory identification, and a significantly lower olfactory identification which started five years preceding MCI diagnosis; 2) olfactory identification continued to decline faster following MCI diagnosis, with a significantly poorer olfactory identification beginning three years before dementia diagnosis; and 3) in dementia-free participants, a steeper decline in olfactory identification was associated with a higher burden of global AD pathology, especially neurofibrillary tangles. Our findings indicate that faster declines in olfactory identification may occur at early stages of dementing disorders, and AD pathologies may underlie the rapid declines in olfactory identification before clinical diagnosis.

In most previous cross-sectional studies, older adults with MCI had a lower olfactory identification score compared to those who were cognitively intact,^4^^,^^5^^,^^7^ although some studies reported only modest or non-statistically significant differences.^32^^,^^33^ Longitudinal studies have also reported impaired olfactory identification in late life in those with incident MCI.^6^ However, more information on olfactory identification trajectories preceding MCI is needed to support its use as a potential screening tool for detecting early stages of cognitive impairment, when the window of opportunity for prevention and treatment maybe still open. To address this, in the present study, we explored patterns of olfactory identification change leading up to the MCI diagnosis. We found that olfactory identification declined faster among individuals who went on to develop MCI compared to those who remained cognitively intact. Notably, the estimated mean level of olfactory identification still fell within the normal range even at the year of MCI diagnosis (i.e., BSIT ≥ 8), though it was significantly poorer starting from five years before MCI diagnosis compared to those who remained cognitively intact. This finding indicates that impaired olfactory identification defined using a single-time test might not be sufficiently sensitive to distinguish individuals with a high risk of MCI from those with normal aging. Thus, monitoring trajectories in olfactory identification may add more predictive value. Moreover, interventions may benefit those with rapid declines in olfactory identification even though they are still within normal identification range and cognitively intact.

Previous cross-sectional and longitudinal studies have reported increased dementia risk^8^^,^^10^^,^^33^^,^^34^ and accelerated progression from MCI to dementia^6^^,^^11^ in those with poorer olfactory identification. We similarly found that, among participants with incident MCI, olfactory identification declined faster for those who went on to develop dementia compared to those who did not. This difference in olfactory identification became apparent beginning three years before dementia diagnosis, with a larger difference closer to the time of dementia diagnosis. These findings suggested that impaired olfactory function may be a predictor of subsequent cognitive impairment and dementia as well as a marker of underlying dementing disorders.

Although olfactory identification has been consistently associated with cognitive impairment, findings regarding its association with brain pathologies are mixed. Several cross-sectional studies have explored the associations of olfactory identification with AD pathologies measured from plasma,^5^ cerebrospinal fluid,^17^ or positron emission tomography.^15^^,^^16^^,^^18^ Some found that amyloid burden was associated with poorer olfactory identification,^15^ whereas others reported only marginal-^16^ or non-significant associations.^5^^,^^17^^,^^18^ Based on Rush MAP, we previously found that both amyloid load and neurofibrillary tangles were inversely associated with olfactory identification.^25^ However, the association between amyloid burden and olfactory identification was attenuated to the point of non-significance after adjusting for tangles, suggesting that tangles may mediate the association of amyloid with olfactory identification.^25^ Previous findings have consistently shown a positive association between neurofibrillary tangles and olfactory dysfunction.^5^^,^^17^^,^^20^

In this study, an increased rate of decline in olfactory identification was separately linked to greater burdens of amyloid beta load and neurofibrillary tangles. After adjusting for each other, the association between decreased olfactory identification and neurofibrillary tangles was slightly attenuated to borderline significance, but the link between olfactory identification declines and amyloid beta load was largely attenuated. Moreover, we found that other AD pathologies, including diffuse and neuritic plaques, were not related to olfactory identification change. These results are consistent with previous clinical-pathological studies reporting that neuritic plaques were less likely to accumulate in the olfactory bulb and olfactory tracts in the early stage of AD progression.^35^^,^^36^ We additionally observed an association between impaired olfactory identification (i.e., BSIT ≤ 8) and the presence of Lewy body pathology starting from at least seven years before death. These results echo previous findings that individuals with Lewy bodies had lower olfactory identification scores.^19^^,^^26^ Taken together, our findings suggest that an accelerated decline in olfactory identification may be indicative of AD pathologies, especially neurofibrillary tangles, and impaired olfactory identification may be related to Lewy body pathology. In sum, our results suggest that monitoring olfactory identification may help to identify older adults who would benefit most from diagnostic testing for these pathologies.

### Strengths and limitations

Strengths of the current study include the longitudinal study design, annual assessments of olfactory identification, relatively long follow-up period, large sample size, and availability of brain autopsy data. However, this study also has several limitations. First, the study population is predominantly White and female with potential self-selection bias, which may reduce the generalizability of our findings to other populations. Second, the 12-item BSIT used in this study may be less sensitive to detect subtle alterations in olfactory identification compared to the 40-item University of Pennsylvania Smell Identification Test, from which it was developed. This may lead to an underestimation of the associations between olfactory identification and dementing disorders. Nevertheless, the 12-item BSIT has a high test-retest reliability coefficient (r = 0.71)^28^ and is more practical than the longer version in clinical settings and large-scale population studies. Third, we examined only olfactory identification and no other olfactory function domains, such as detection threshold and sensitivity, due to substantial missing information for those variables. However, olfactory identification is more strongly correlated with neuropsychological measures than the other two domains of olfactory function.^37^ Fourth, olfactory identification requires participants to compare their olfactory experience with information stored in memory in addition to detecting or discriminating odors. It is reasonable to speculate that olfactory identification may be reversely impacted by severe cognitive impairment. This point is also supported by our results showing that the difference in olfactory identification between dementia and non-dementia cases became larger closer to the time of dementia diagnosis. Therefore, we should interpret the associations between olfactory identification and dementing disorders with caution, considering its role as a predictor as well as a marker of cognitive impairment. Fifth, the non-significant association between Lewy body pathology and olfactory identification change should be interpreted with caution. A faster decline in relation to Lewy body pathology may occur earlier and not be captured by seven years preceding death. Future studies with a longer follow-up before death and with sufficient sample size are warranted to explore the role of Lewy body pathology in relation to olfactory identification change. Sixth, in the Rush MAP, although a clinical diagnosis of cognitive status was rendered annually, incident MCI or dementia may occur at any time during adjacent follow-up assessments. However, this would likely lead to only a slight underestimation of our results. Finally, in this study, global AD pathologies (including neuritic plaques, diffuse plaques, and neurofibrillary tangles) were determined from five regions, including the midfrontal cortex, midtemporal cortex, inferior parietal cortex, entorhinal cortex, and hippocampus. Brain pathologies in other parts of the central olfactory system, such as the olfactory bulbs and piriform cortex, were not available. However, evidence has shown that those regions develop neurofibrillary tangles in earlier stages (i.e., Braak’s stage 0 and 1) than other parts of the central olfactory system.^38^ Missing information on these earlier damaged parts of central olfactory system (e.g., olfactory bulbs) may lead to an underestimation of the associations between brain pathologies and olfactory identification, given that the lowest burden group in this study may include participants with high neurofibrillary tangles in those unmeasured brain regions. Future studies examining brain pathologies in these regions are warranted to better understand the mechanisms underlying olfactory identification changes.

### Conclusions

The present findings provide evidence of faster declines in olfactory identification preceding incident MCI and dementia. Our results also indicate that a high burden of global AD pathology, especially neurofibrillary tangles, may underlie accelerated olfactory identification decline. Monitoring olfactory identification changes may be a strategy for early detection of individuals at a high risk of developing MCI or dementia, even among those with olfactory identification within the normal range. Future studies are warranted to explore whether interventions or treatments can prevent or delay the progression of dementing disorders when administered early enough, such as among individuals with rapid olfactory decline but no apparent cognitive impairment.

## Contributors

JG and WX conceived the research hypothesis. JG performed the data analyses and drafted the initial manuscript. JG and JW accessed and verified the underlying data reported in the manuscript. WX and DB obtained funding and supervised the study. All authors contributed to the interpretation of the results and critically revised the manuscript. All authors read and approved the final version of the manuscript.

## Data sharing statement

Rush MAP data can be requested at https://www.radc.rush.edu. All SAS codes can be requested via correspondence email.

## Declaration of interests

All authors have completed the ICMJE uniform disclosure form. All authors report no disclosures relevant to the manuscript.
