# Supplementary material for: Trajectories of olfactory identification preceding incident mild cognitive impairment and dementia: a longitudinal study
Source: eBioMedicine. 2023 Oct 28;98:104862. doi: 10.1016/j.ebiom.2023.104862 (PMC10628348; doi:10.1016/j.ebiom.2023.104862)
Supplement: Supplementary Text S1, Figs. S1–S3, and Tables S1–S6 [file mmc1.docx]

**Supplementary files**

**Supplementary Text 1. Data collection**

Education was recorded as the duration of formal schooling in years. Physical activity was expressed as total number of hours per week that participants engaged in 5 types of activities (i.e., gardening or yard work, walking for exercise, bicycle riding, calisthenics or general exercise, and swimming or water exercise) within the past 2 weeks using questions adapted from the National Health Interview Survey.^1^

Diabetes was ascertained based on fasting plasma glucose ≥ 126 mg/dl, glycated haemoglobin (HbA_1c_) ≥ 6.5%, random blood glucose ≥ 200 mg/dl, the use of anti-diabetic medication, or self-reported medical history.^2^ Hypertension was identified based on systolic and diastolic blood pressure ≥140/90 mmHg or self-reported history of hypertension. Vascular diseases, including stroke, claudication, heart conditions (i.e., heart attack or coronary, myocardial infarction, coronary occlusion, or coronary thrombosis), and congestive heart failure, were self-reported by participants. In addition to self-report, stroke was also ascertained based on clinical and neurological examination, and cognitive testing.

Blood samples were collected for all participants at study entry. Participants were genotyped for apolipoprotein E (*APOE*) alleles by Polymorphic DNA Technologies and were dichotomized as ε4 carriers vs. non-carriers. Further details about the data collection are available at the Rush Alzheimer’s Disease Center Resource Sharing Hub (www.radc.rush.edu).


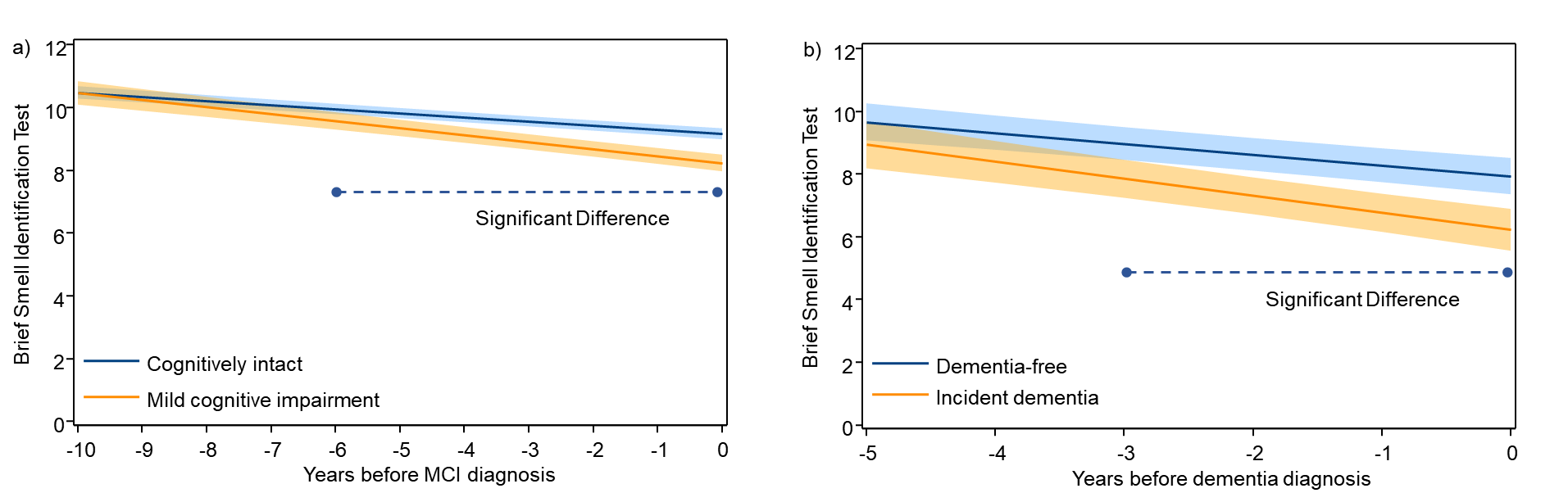


**Supplementary Figure 1. Trajectories of Brief Smell Identification Test (BSIT) in the 10 years before mild cognitive impairment (MCI) diagnosis (a) and in the 5 years before dementia diagnosis (b)**

The figure represents marginal effects of MCI on trajectories of BSIT, adjusted for age at time 0, sex, and education. For the trajectory before MCI, we used the baseline cognitively intact sample and included 619 participants with ≥3 repeated BSIT measurements. For the trajectory before dementia, we used the incident MCI sample and included 96 participants with ≥3 repeated BSIT measurements. The band represents the 95% confidence interval of estimated mean BSIT. For the multiple testing and comparisons, the significant differences were defined using adjusted *P* value <0.05 calculated from a simulation-based approach combined with a step-down fashion. For BSIT trajectories before MCI diagnosis (a), β coefficient for MCI × time is -0.09 (95% CI -0.14, -0.05; *P* <0.001). For BSIT trajectories before dementia diagnosis (b), β coefficient for dementia × time is -0.20 (95% CI -0.39, -0.01; *P* = 0.043).

**Supplementary Table 1. Differences in Brief Smell Identification Test (BSIT) between mild cognitive impairment (MCI) cases and cognitively intact participants in the 10 years before MCI diagnosis in cognitively intact sample**

| **Year** | **No. of cognitively intact participants** | **No. of MCI participants** | **Incident MCI vs. cognitively intact** | |
| --- | --- | --- | --- | --- |
|  |  |  | **Difference in mean (95% CI)** | ***P*-value** |
| -10 | 116 | 11 | -0.01 (-0.49, 0.48) | 0.978 |
| -9 | 174 | 21 | -0.10 (-0.55, 0.35) | 0.631 |
| -8 | 209 | 33 | -0.19 (-0.61, 0.22) | 0.315 |
| -7 | 238 | 53 | -0.29 (-0.67, 0.10) | 0.108 |
| -6 | 267 | 67 | -0.38 (-0.74, -0.02) | 0.023 |
| -5 | 302 | 90 | -0.47 (-0.81, -0.13) | 0.002 |
| -4 | 366 | 104 | -0.57 (-0.89, -0.24) | <0.001 |
| -3 | 404 | 144 | -0.66 (-0.98, -0.33) | <0.001 |
| -2 | 411 | 162 | -0.75 (-1.08, -0.42) | <0.001 |
| -1 | 394 | 161 | -0.85 (-1.18, -0.51) | <0.001 |
| 0 | 441 | 178 | -0.94 (-1.29, -0.58) | <0.001 |

Difference in mean was calculated as the mean of BSIT in participants with MCI minus that in those cognitively intact. Negative value means that olfactory identification was lower in participants with MCI. Model was adjusted for age at time 0, sex, and education.

There were 619 participants with ≥3 repeated BSIT measurements before MCI onset included for this analysis.

**Supplementary Table 2. Differences in Brief Smell Identification Test (BSIT) between incident dementia cases and participants who remained mild cognitive impairment (MCI) non-dementia ones in the 5 years before dementia diagnosis in the incident MCI sample**

| **Year** | **No. of non-dementia participants** | **No. of dementia participants** | **Incident dementia vs. remain MCI** | |
| --- | --- | --- | --- | --- |
|  |  |  | **Difference in mean (95% CI)** | ***P*-value** |
| -5 | 31 | 15 | -0.72 (-1.80, 0.35) | 0.128 |
| -4 | 44 | 22 | -0.92 (-1.88, 0.04) | 0.037 |
| -3 | 59 | 28 | -1.12 (-2.01, -0.22) | 0.007 |
| -2 | 58 | 33 | -1.31 (-2.19, -0.44) | 0.001 |
| -1 | 53 | 31 | -1.51 (-2.42, -0.60) | <0.001 |
| 0 | 62 | 34 | -1.71 (-2.70, -0.71) | <0.001 |

Difference in mean was calculated as the mean of BSIT in participants with dementia minus that in those remain MCI. Negative value means that olfactory identification was lower in participants with dementia. Model was adjusted for age at time 0, sex, and education.

There were 96 participants with ≥3 repeated BSIT measurements before dementia onset included for this analysis.

**Supplementary Table 3. β coefficient and 95% confidence interval (CI) for the association of Brief Smell Identification Test (BSIT) trajectories with brain pathologies before death among dementia-free participants**

| **Brain pathology** | **β coefficient (95% CI)** | ***P* value** |
| --- | --- | --- |
| **Global AD pathology** |  |  |
| Intercept | 12.81 (9.25, 16.37) | <.0001 |
| Pathology (T2 vs. T1) | 0.09 (-0.51, 0.69) | 0.769 |
| Pathology (T3 vs. T1) | -0.81 (-1.42, -0.2) | 0.010 |
| Time | -0.19 (-0.26, -0.12) | <.0001 |
| Pathology * Time (T2 vs. T1) | -0.07 (-0.17, 0.03) | 0.174 |
| Pathology * Time (T3 vs. T1) | -0.16 (-0.28, -0.04) | 0.011 |
| **Neurofibrillary tangles** |  |  |
| Intercept | 12.56 (9.01, 16.1) | <.0001 |
| Neurofibrillary tangles (T2 vs. T1) | -0.48 (-1.09, 0.12) | 0.118 |
| Neurofibrillary tangles (T3 vs. T1) | -1.2 (-1.81, -0.58) | 0.000 |
| Time | -0.19 (-0.26, -0.12) | <.0001 |
| Neurofibrillary tangles * Time (T2 vs. T1) | -0.07 (-0.18, 0.03) | 0.167 |
| Neurofibrillary tangles * Time (T3 vs. T1) | -0.15 (-0.26, -0.04) | 0.009 |
| **Neuritic plaque** |  |  |
| Intercept | 13.08 (9.53, 16.62) | <.0001 |
| Pathology (T2 vs. T1) | 0.08 (-0.52, 0.68) | 0.788 |
| Pathology (T3 vs. T1) | -0.67 (-1.28, -0.06) | 0.031 |
| Time | -0.2 (-0.27, -0.13) | <.0001 |
| Pathology * Time (T2 vs. T1) | -0.08 (-0.18, 0.03) | 0.165 |
| Pathology * Time (T3 vs. T1) | -0.11 (-0.22, 0.01) | 0.062 |
| **Diffuse plaque** |  |  |
| Intercept | 12.94 (9.32, 16.57) | <.0001 |
| Pathology (T2 vs. T1) | 0.13 (-0.47, 0.74) | 0.662 |
| Pathology (T3 vs. T1) | -0.47 (-1.08, 0.14) | 0.134 |
| Time | -0.21 (-0.28, -0.14) | <.0001 |
| Pathology * Time (T2 vs. T1) | -0.05 (-0.16, 0.05) | 0.313 |
| Pathology * Time (T3 vs. T1) | -0.1 (-0.22, 0.01) | 0.082 |
| **Amyloid beta load** |  |  |
| Intercept | 12.33 (8.12, 16.53) | <.0001 |
| Pathology (T2 vs. T1) | 0 (-0.7, 0.69) | 0.992 |
| Pathology (T3 vs. T1) | -0.82 (-1.55, -0.08) | 0.030 |
| Time | -0.23 (-0.33, -0.14) | <.0001 |
| Pathology * Time (T2 vs. T1) | -0.12 (-0.26, 0.01) | 0.073 |
| Pathology * Time (T3 vs. T1) | -0.21 (-0.38, -0.04) | 0.014 |
| **Vascular disease pathology** |  |  |
| Intercept | 13.98 (10.37, 17.59) | <.0001 |
| Pathology (T2 vs. T1) | 0.28 (-0.35, 0.91) | 0.380 |
| Pathology (T3 vs. T1) | 0.27 (-0.32, 0.85) | 0.372 |
| Time | -0.2 (-0.27, -0.14) | <.0001 |
| Pathology * Time (T2 vs. T1) | -0.1 (-0.21, 0.02) | 0.100 |
| Pathology * Time (T3 vs. T1) | -0.07 (-0.18, 0.03) | 0.152 |
| **Gross chronic infarcts** |  |  |
| Intercept | 13.21 (9.65, 16.76) | <.0001 |
| Pathology (Present vs. Absent) | -0.29 (-0.81, 0.22) | 0.263 |
| Time | -0.24 (-0.3, -0.18) | <.0001 |
| Pathology * Time (Present vs. Absent) | -0.03 (-0.13, 0.06) | 0.486 |
| **Chronic microinfarcts** |  |  |
| Intercept | 13.19 (9.63, 16.75) | <.0001 |
| Pathology (Present vs. Absent) | 0.38 (-0.14, 0.9) | 0.147 |
| Time | -0.3 (-0.35, -0.24) | <.0001 |
| Pathology * Time (Present vs. Absent) | 0.12 (0.02, 0.21) | 0.014 |
| **Lewy body pathology** |  |  |
| Intercept | 13.47 (10.12, 16.83) | <.0001 |
| Pathology (Present vs. Absent) | -1.98 (-2.63, -1.33) | <.0001 |
| Time | -0.26 (-0.3, -0.21) | <.0001 |
| Pathology * Time (Present vs. Absent) | 0.03 (-0.10, 0.16) | 0.637 |

Abbreviations: T1: lowest tertile; T2: middle tertile; T3: highest tertile.

There were 249 participants with ≥2 repeated BSIT measurements before death included for this analysis. Model was adjusted for age at time 0, sex, and education.

**Supplementary Table 4. Differences in Brief Smell Identification Test (BSIT) between participants with and without Lewy-body pathology in the 7 years before death in dementia-free participants**

| **Year** | **No. of absence of Lewy-body pathology** | **No. of presence of Lewy-body pathology** | **Presence vs. absence** | |
| --- | --- | --- | --- | --- |
|  |  |  | **Difference in mean (95% CI)** | ***P*-value** |
| -7 | 63 | 10 | -2.20 (-3.21, -1.19) | <0.001 |
| -6 | 88 | 14 | -2.17 (-3.07, -1.27) | <0.001 |
| -5 | 96 | 16 | -2.14 (-2.95, -1.32) | <0.001 |
| -4 | 126 | 21 | -2.10 (-2.85, -1.36) | <0.001 |
| -3 | 156 | 26 | -2.07 (-2.77, -1.38) | <0.001 |
| -2 | 192 | 29 | -2.04 (-2.73, -1.36) | <0.001 |
| -1 | 226 | 38 | -2.01 (-2.72, -1.30) | <0.001 |
| 0 | 254 | 47 | -1.98 (-2.74, -1.22) | <0.001 |

Difference in mean was calculated as the mean of BSIT in participants with Lewy-body pathology minus that in those without Lewy-body pathology. Negative value means that olfactory identification was lower in participants with Lewy-body pathology. Model was adjusted for age at time 0, sex, and education.


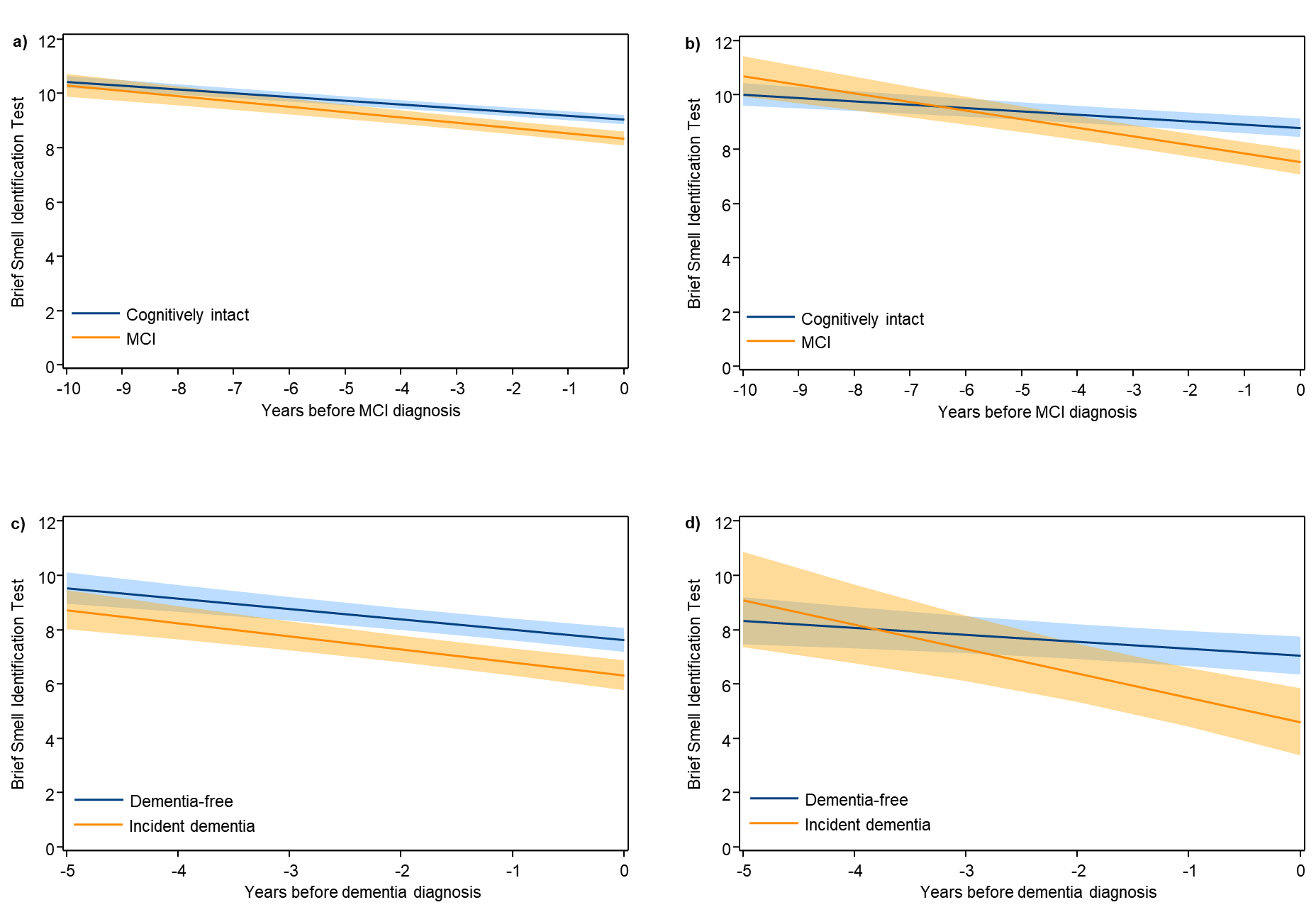


**Supplementary Figure 2. Trajectories of Brief Smell Identification Test (BSIT) in the 10 years before mild cognitive impairment (MCI) diagnosis in female (a) and male (b)**

The figure represents marginal effects of MCI or dementia on trajectories of BSIT, adjusted for age at time 0 and education. The band represents the 95% confidence interval of estimated mean BSIT. For BSIT trajectories before MCI diagnosis, β coefficient for MCI × time is -0.06 (95% CI -0.11, -0.01; *P* = 0.020) in female (a) and -0.19 (95% CI -0.28, -0.11; *P* <0.001) in male (b).

**Supplementary Table 5. Differences in Brief Smell Identification Test (BSIT) between mild cognitive impairment (MCI) cases and cognitively intact participants in the 10 years before MCI diagnosis in cognitively intact sample by sex**

| **Year** | **Among female** | |  | **Among male** | |
| --- | --- | --- | --- | --- | --- |
|  | **Difference in mean (95% CI)** | ***P*-value** |  | **Difference in mean (95% CI)** | ***P*-value** |
| -10 | -0.13 (-0.69, 0.42) | 0.579 |  | 0.67 (-0.32, 1.65) | 0.162 |
| -9 | -0.19 (-0.70, 0.32) | 0.405 |  | 0.47 (-0.43, 1.38) | 0.293 |
| -8 | -0.25 (-0.72, 0.22) | 0.244 |  | 0.28 (-0.55, 1.11) | 0.489 |
| -7 | -0.31 (-0.74, 0.12) | 0.122 |  | 0.08 (-0.68, 0.84) | 0.799 |
| -6 | -0.36 (-0.76, 0.03) | 0.048 |  | -0.11 (-0.81, 0.59) | 0.749 |
| -5 | **-0.42 (-0.79, -0.05)** | **0.014** |  | -0.30 (-0.96, 0.35) | 0.341 |
| -4 | **-0.48 (-0.83, -0.13)** | **0.003** |  | -0.50 (-1.11, 0.12) | 0.098 |
| -3 | **-0.54 (-0.88, -0.20)** | **0.001** |  | **-0.69 (-1.29, -0.10)** | **0.014** |
| -2 | **-0.59 (-0.93, -0.25)** | **0.000** |  | **-0.89 (-1.48, -0.29)** | **0.001** |
| -1 | **-0.65 (-1.00, -0.30)** | **0.000** |  | **-1.08 (-1.69, -0.47)** | **0.000** |
| 0 | **-0.71 (-1.07, -0.34)** | **0.000** |  | **-1.28 (-1.92, -0.63)** | **0.000** |

Difference in mean was calculated as the mean of BSIT in participants with MCI minus that in those cognitively intact. For the multiple testing and comparisons, the significant differences were defined using adjusted *P* value <0.05 calculated from a simulation-based approach combined with a step-down fashion. Negative value means that olfactory identification was lower in participants with MCI. Model was adjusted for age at time 0 and education.


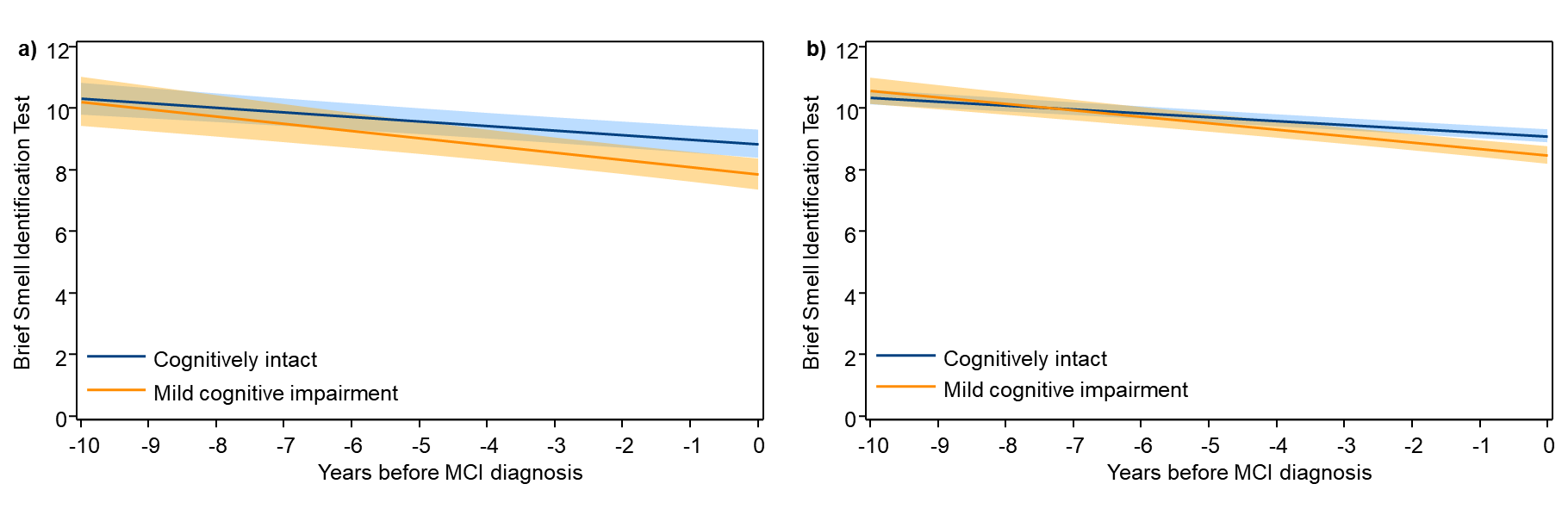


**Supplementary Figure 3. Trajectories of Brief Smell Identification Test (BSIT) in the 10 years before mild cognitive impairment (MCI) diagnosis in apolipoprotein E (APOE) ε4 carriers (a) and non-carriers (b)**

The figure represents marginal effects of MCI or dementia on trajectories of BSIT, adjusted for age at time 0, sex, and education. The band represents the 95% confidence interval of estimated mean BSIT. For BSIT trajectories before MCI diagnosis, β coefficient for MCI × time is -0.10 (95% CI -0.19, 0.00; *P* = 0.051) in *APOE* ε4 carriers (a) and -0.08 (95% CI -0.13, -0.03; *P* <0.001) in non-carriers (b).

**Supplementary Table 6. Differences in Brief Smell Identification Test (BSIT) between mild cognitive impairment (MCI) cases and cognitively intact participants in the 10 years before MCI diagnosis in cognitively intact sample by apolipoprotein E (APOE) genotype**

| **Year** | **Among *APOE* ε4 carriers** | |  | **Among *APOE* ε4 non-carriers** | |
| --- | --- | --- | --- | --- | --- |
|  | **Difference in mean (95% CI)** | ***P*-value** |  | **Difference in mean (95% CI)** | ***P*-value** |
| -10 | -0.04 (-1.13, 1.05) | 0.926 |  | 0.22 (-0.33, 0.77) | 0.445 |
| -9 | -0.14 (-1.15, 0.87) | 0.774 |  | 0.13 (-0.37, 0.64) | 0.593 |
| -8 | -0.23 (-1.17, 0.70) | 0.606 |  | 0.05 (-0.42, 0.52) | 0.833 |
| -7 | -0.33 (-1.19, 0.53) | 0.436 |  | -0.03 (-0.47, 0.40) | 0.864 |
| -6 | -0.43 (-1.23, 0.38) | 0.281 |  | -0.12 (-0.52, 0.29) | 0.589 |
| -5 | -0.52 (-1.28, 0.24) | 0.162 |  | -0.20 (-0.58, 0.18) | 0.311 |
| -4 | -0.62 (-1.34, 0.11) | 0.086 |  | -0.28 (-0.65, 0.08) | 0.120 |
| -3 | -0.71 (-1.42, 0.00) | 0.042 |  | -0.37 (-0.72, -0.01) | 0.037 |
| -2 | -0.81 (-1.52, -0.09) | 0.021 |  | -0.45 (-0.81, -0.09) | 0.009 |
| -1 | -0.90 (-1.64, -0.17) | 0.012 |  | -0.53 (-0.90, -0.16) | 0.003 |
| 0 | -1.00 (-1.77, -0.23) | 0.008 |  | -0.61 (-1.00, -0.23) | 0.001 |

Difference in mean was calculated as the mean of BSIT in participants with MCI minus that in those cognitively intact. For the multiple testing and comparisons, the significant differences were defined using adjusted *P* value <0.05 calculated from a simulation-based approach combined with a step-down fashion. Negative value means that olfactory identification was lower in participants with MCI. Model was adjusted for age at time 0, sex, and education.

**References**

1 1985 Health Interview Survey. National Center for Health Statistics, Series 10. 1985: 86–1568.

2 Association AD. 2. Classification and Diagnosis of Diabetes: Standards of Medical Care in Diabetes—2021. *Diabetes Care* 2021; **44**: S15–33.
